# Childhood Asthma and Community Resilience

**DOI:** 10.1002/ppul.71513

**Published:** 2026-02-16

**Authors:** Maureen Y. Lichtveld, Kaitlin Kirkpatrick Heimke, Laura J. Dietz, Firoz Abdoel Wahid, Jeanine M. Buchanich, Brian Conner Earle, Terry L. Noah

**Affiliations:** ^1^ University of Pittsburgh School of Public Health Pittsburgh Pennsylvania USA; ^2^ University of Pittsburgh School of Health and Rehabilitation Sciences Pittsburgh Pennsylvania USA; ^3^ University of North Carolina School of Medicine North Carolina USA

**Keywords:** environment, pediatrics, public health, respiratory health

## Abstract

**Background:**

Pediatric asthma remains a leading global health concern and disproportionately affects children in low‐income and environmentally burdened communities. Chemical and non‐chemical stressors, including air pollution, allergens, climate‐related events, and psychosocial stress, contribute to asthma exacerbations and hinder effective disease management.

**Objectives:**

This paper explores the intersection of childhood asthma and community resilience, emphasizing the influence of environmental exposures, public health infrastructure, and targeted interventions on pediatric respiratory outcomes.

**Methods:**

A series of case studies is used to examine how environmental and social stressors shape asthma risk and management. These case studies highlight patterns of exposure, community‐level challenges, and the role of public health systems and community‐engaged strategies.

**Results:**

Findings illustrate that combined environmental burdens and insufficient public health infrastructure contribute to persistent asthma disparities. Case studies also show that communities with stronger resilience—through resources, engagement, or adaptive systems—experience improved support for children with asthma.

**Conclusions:**

Longitudinal research, policy reform, and community‐engaged interventions are essential to mitigate asthma disparities and support children's health in the face of environmental adversity.

## 1 Background

Children are especially vulnerable to environmental exposures that influence lung health, making pediatric respiratory diseases a critical public health challenge.

The global burden of pediatric asthma in 2021 was estimated at approximately 4314 cases per 100,000 children and adolescents under 20 years, with the highest prevalence among school‐aged children (5–9 years) at approximately 5427 per 100,000. In high‐income regions such as North America, prevalence among children aged 5–9 years reaches as high as 16,553 per 100,000 [1]. These figures translate to millions of affected children worldwide, with substantial geographical and socioeconomic disparities in both prevalence and disease management.

Environmental triggers contributing to pediatric asthma include outdoor pollutants (PM2.5, nitrogen dioxide, ozone), indoor allergens (dust mites, mold, cockroaches, pet dander), and chemical irritants such as household cleaners and pesticides. These exposures are compounded by non‐chemical stressors, including viral infections (respiratory syncytial virus, influenza, COVID‐19), psychological stress, and climate‐related events such as wildfires, all of which exacerbate respiratory symptoms and disease progression [2, 3, 4, 5]. Notably, children in urban, low‐income, and minority communities face disproportionate exposure to these hazards. Housing conditions, poor ventilation, and limited access to healthy food and green space further magnify this burden [3].

Pediatric asthma remains a leading cause of emergency visits, hospitalizations, and school absences worldwide, creating a profound health and economic toll on families and communities [1, 2]. For families, treated cases among children and adolescents add more than $3300 in annual medical expenses, while hospitalizations alone accounted for an estimated $480.6 million in total US costs in 2022 [6, 7].

Addressing this challenge requires improving indoor and outdoor air quality, reducing exposures to known triggers, and strengthening community resilience through targeted public health policies. Equally important are efforts to foster environmental health literacy among caregivers and to integrate climate adaptation strategies into pediatric healthcare, helping protect vulnerable children against both current and emerging environmental threats [8].

The prevalence of environmentally driven lung disease in children underscores the urgent need for coordinated action by healthcare providers, policymakers, and communities to reduce exposures and improve outcomes. Ongoing research and surveillance remain essential to guide prevention efforts and build environmental resilience in pediatric populations.

## 2 Case Studies

**Figure 1:**
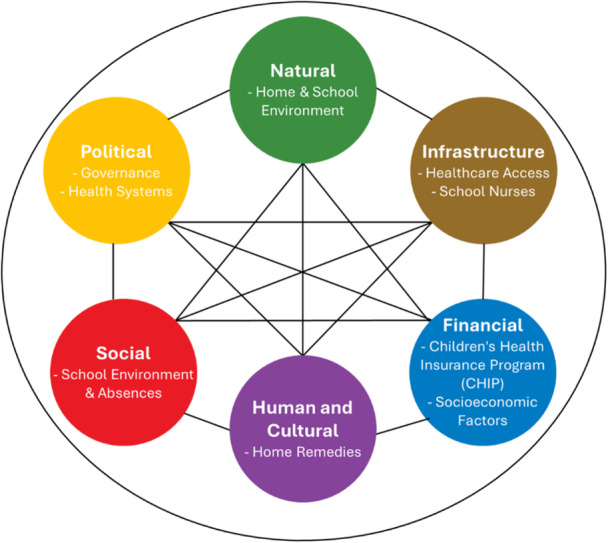
Six capitals of community resilience.

The burden of childhood asthma has been documented worldwide. Studies have addressed exposures to asthma triggers and exacerbations of existing moderate to severe asthma in children as young as 4 years old. While genetic predisposition does play a role, the negative consequences of environmental exposures are well documented. At the same time, numerous studies also examined the impact of interventions, especially those involving asthma counselors, often in combination with mitigation of asthma triggers. These interventions are most successful in communities with sustained capital resources. These capitals represent the domains of community resilience [9]. Of the six capitals—natural, human, social, financial, political, and infrastructure (Figure 1)—the greatest challenge faced by childhood asthma interventions is in communities with a lack of a functioning public health infrastructure. Access to and quality of primary care, as well as socio‐economic capacity to address in‐home asthma triggers, are key indicators of public health infrastructure. The case studies highlighted below illustrate several aspects of exposures, impact, and challenges associated with childhood asthma to achieve sustained resilience at the community‐level.

### 2.1 Case Study: Head‐Off Environmental Asthma in Louisiana (HEAL) Study

Background: On August 29, 2005, Hurricane Katrina hit New Orleans and flooded 80% of the City of New Orleans for almost 6 weeks. The magnitude of mold spores and other asthma triggers was the impetus for the development of the Head of Environmental Asthma in Louisiana (HEAL) study. The study represents a unique environmental‐ and asthma counselor hybrid intervention that addressed a two‐pronged objective: to examine the relationships between the post‐Katrina environment and childhood asthma and determine the impact of an asthma counselor intervention aimed at mitigating in‐home asthma triggers [10].

Methods: Annually, 4–12‐year‐old children with moderate‐to‐severe asthma received two clinical evaluations, three home environmental evaluations, and the asthma intervention. The study examined 4 end points quarterly: symptom days, medication use, and unscheduled emergency department or clinic visits. From design to dissemination, a community advisory group representing school principals, the New Orleans Department of Health, and community organizations guided the activities.

Findings: 182 children were enrolled from households with annual incomes < $15,000. HEAL children were symptomatic, averaging 6.6 symptom days in the 2 weeks before participating in HEAL's baseline assessments. 76% of the children had at least one unscheduled visit in the preceding 3 months.

Environmental home assessments consisted of indoor air analyses and dust collection. More than half (62%) of the children were living in homes that had been damaged by rain, flooding, or both. Of note is that the Alternaria antigen was detected in dust from 98% of homes. Concentration of other mold spores was lower than in other studies, possibly because some children moved into new or clean homes, including temporary trailers, when they returned to New Orleans [11].

Immunological assessments showed that 89% of the children tested positive to ≥1 indoor allergen. Allergen‐specific sensitivities ranged from 18% to 67% [12]. Asthma symptom days did not differ with skin test sensitivity. A seemingly counterintuitive finding was that increased symptoms were observed in children whose baseline indoor airborne mold concentrations were below median levels. There are several potential explanations for this discrepancy: the majority of children in the HEAL study tested positive for more than one allergen. The indoor environmental assessments identified several asthma triggers beyond mold, including cat‐, mouse‐, and dog dander and dust mites. The environmental conditions in the immediate Post Katrina period varied considerably, resulting in challenging exposure characterization scenarios—some families lived in trailers next to the damaged homes and were involved in clean‐up activities, while others moved into trailers where building material off‐gassing was still ongoing. However, the challenging environmental and social‐economic circumstances of these children likely played a key role in their symptomatology and can potentially explain this result; For example, the second highest association with asthma exacerbation was losing a pet or changing school more than twice in one school year.

Asthma counselor intervention: In addition to the home environmental assessments, HEAL embedded a community‐engaged asthma intervention. Early in the intervention, it became clear that significant tailoring was needed to increase participation. Specifically, parents were more interested in the environmental assessment than in the asthma counseling (AC) sessions. By combining the sharing of the environmental assessment results with the counseling session, the AC visit rate increased by 92.3%. Significant improvements were observed across several adherence measures. This adaptation may inform AC intervention in other resilience‐ challenged communities [13].

### 2.2 Case Study: Fracking and Asthma Exacerbations

Background: The use of unconventional natural gas development (UNGD)—commonly known as fracking—has expanded in Southwestern Pennsylvania in recent years. Previous research conducted in Eastern Pennsylvania has identified significant associations between each phase of the fracking process and exacerbations of asthma symptoms [14, 15]. Therefore, Buchanich et al. aimed to replicate and expand on this research in a Southeastern Pennsylvania cohort by characterizing the effect of fracking use upon asthma exacerbations of multiple severity levels across varying distances of exposure [16].

Methods: The cohort consisted of 46,676 asthma patients aged 5−90 collected from University of Pittsburgh Medical Center electronic medical records from 2011 to 2020. Three categories of asthma exacerbation were used: severe exacerbation (i.e., initiation or increase of systemic corticosteroid medications), exacerbations requiring an emergency department visit, and exacerbations requiring hospitalization. Exposure to fracking was measured across four distances from exposure ranging from 1 to 10 miles for each of the four phases of fracking: well pad preparation, drilling, hydraulic fracturing, and production.

Findings: Buchanich et al. found compelling evidence linking fracking to asthma exacerbations [16]. After controlling for confounding, they found strong evidence for an increased risk specifically during the production phase for residences located within 1−10 miles. This risk was consistently found for severe exacerbations, emergency department visits, and hospitalizations based on consistent statistically significantly elevated odds ratios. Elevations ranged from 2 to 8 times the baseline of no wells within 10 miles of the patient's residence.

This information should be considered in determining risk communication and assessment for these vulnerable populations, particularly during the production phase. These findings have prompted calls for increased setback distances between fracking sites and residential areas, especially schools and homes. While the studies themselves focus primarily on health outcomes, the broader discussion around resilience involves:

- Community advocacy: Residents pushed for these studies due to unexplained health issues, demonstrating civic resilience.
- Policy response: Proposals to increase buffer zones reflect institutional resilience in adapting to emerging health threats.
- Scientific inquiry: The research itself is a form of resilience, responding to public concern with rigorous investigation.

### 2.3 Case Study: The East Palestine Train Derailment

Background: On February 3, 2023, a Norfolk Southern freight train carrying hazardous materials derailed in East Palestine, Ohio, prompting evacuations and shelter‐in‐place orders. The Environmental Protection Agency (EPA) confirmed the release of vinyl chloride (VC), butyl acrylate, ethylhexyl acrylate, and ethylene glycol monobutyl ether into the air, surface soil, and surface waters. On February 6, a controlled burn was conducted to prevent explosions, releasing phosgene gas and hydrochloric acid. Phosgene, produced by combustion of VC, is a poisonous chlorine‐containing gas that was used as a chemical warfare agent during World War 1, and is extremely toxic by short‐term acute exposure, producing severe lung injury characterized by pulmonary edema, severe tissue injury, and death [17]. Inhalation of hydrochloric acid fumes can similarly produce acute lung injury [18]. On February 8, the mandatory evacuation order was lifted, and people were informed that it was “safe to return” to their homes and that air and water testing performed by Incident Command revealed “nothing of concern.”

Childhood asthma was already a significant concern in East Palestine and the surrounding counties. According to the 2021 Ohio Asthma Burdens Report, children under age 5 had the highest rate of inpatient asthma hospitalizations, more than twice the rate of adults aged 18–34. Nearly 20% of Ohio children missed 1 to 4 days of school due to asthma, and 9.4% missed 5 or more days [19]. Columbiana and Mahoning Counties were designated as Asthma Priority Counties in 2020 due to elevated rates of childhood asthma and hospitalizations. In Pennsylvania, childhood asthma rates were 8.6%, exceeding the national average of 6.7%, with Beaver County reporting 8.61% between 2020 and 2021.

Methods: In response to the derailment, the National Institute of Environmental Health Sciences (NIEHS) funded six research projects, including one led by University of Pittsburgh investigators, *East Palestine Community‐Engaged Environmental Exposure, Health Data, and Biospecimen Bank*. Aim 3 of this study is focused on collecting baseline psychosocial data from parents regarding their children, utilizing the Health Anxiety by Proxy Scale (HAPYS) and a Child Health Survey comprised of the CSI‐24 and the Brief Environmental Exposure and Sensitivity Inventory (BREESI) [20, 21, 22].

Findings: Preliminary survey data from 11 parents (reporting on 17 children, mean age 8.78) revealed that 65% of children experienced respiratory symptoms such as breathing problems, congestion, and frequent infections. Of these, 73% were evaluated by medical professionals, and 55% continued to experience symptoms. Additional health issues included rashes (41%), musculoskeletal pain (41%), stomach pain (35%), and eye irritation (29%). Mental health impacts were also significant. Parents reported anxiety (47%) and depressed mood (41%) in children post‐disaster, with continued symptoms in 88% and 86% of affected children, respectively. Despite this, mental health services were rarely accessed. Parents expressed high levels of anxiety about their children's health (91%), with 50% reporting “a lot” or “a whole lot” of concern. Many worried about undetected serious illness (72.8%) and felt sadness (81.8%) and guilt (54.5%) over their ongoing concerns, often limiting children's outdoor activities due to safety fears.

This case underscores the vulnerability of children to environmental disasters and the importance of resilience. Children and youth are reported to be disproportionately affected by disasters, but their responses and needs have only recently begun to be understood and considered in disaster‐related planning [23]. Children and youth comprise uniquely vulnerable groups in response to environmental toxic exposures based on both biological and developmental factors [24, 25]. Children and youth have increased metabolic and oxygen consumption rates as compared to adults, heightening potential absorption of air‐borne toxins; their smaller size and tissue mass may also increase children's concentration of chemical toxins, and they are often closer to environmental pathways affected by chemical toxins, such as water and soil, than adults. The derailment represents an adverse childhood event with lasting physical and psychological consequences. Addressing these challenges requires coordinated public health responses, mental health support, and community engagement to build resilience and mitigate long‐term impacts.

## 3 How to Create Resilience: Implications for Science, Policy, and Practice

### 3.1 Research Considerations for Resilience

The health resilience of children with asthma could be strengthened through a better understanding of the biological pathways affected by environmental changes. Research with experimental models has identified multiple pathways for triggering asthma exacerbations. These include direct inflammatory effects of particulate pollutants in the airways via oxidative stress and epithelial barrier disruption, recently reviewed in detail by Bowman et al. [26]. Climate‐related changes in allergen quantity or composition or may also impact the course of asthmatic airway inflammation. For example, evidence has been found that pollutant‐driven oxidative and nitrative modifications of pollen surface proteins can increase their allergenic potential [27, 28]. Jaspers and coworkers recently reported a study in a cohort of volunteers exposed to Canadian wildfire smoke in June 2023; while all participants wore masks while outdoors, exposures still increased allergic biomarkers in the nasal mucosa, suggesting that gas‐phase components of wildfire smoke may contribute to respiratory allergic inflammation [29].

Controlled, short experimental exposures in human volunteers have proven to be a safe and informative approach for the investigation of health effects of air pollutants, including woodsmoke [30, 31, 32, 33, 34]. This line of research is not typically considered appropriate for participation by children, but data from studies in young adult volunteers are likely directly relevant to children and adolescents.

It is likely that environmental challenges related to a warming climate will persist or worsen in the coming decades. Thus, another important research focus is on mitigation strategies that could be applied immediately and on a large scale. These strategies include improved personal pollutant and allergen monitoring systems [35, 36, 37] and affordable home air‐filtration systems [38]. For asthma in particular, a clear short‐term research need is to test the effectiveness of existing treatments to mitigate health effects from climate‐driven environmental exposures [39, 40], as well as a reduction in the use of aerosol propellants, which contribute to greenhouse gases [41].

From an environmental epidemiology perspective, Sandifer et al. proposed a community health observing system illustrating not only the importance of data collection during the “inter‐disaster” period but also describing a systematic and cost‐effective approach to long‐term environmental epidemiologic studies. The proposed design embeds the use of existing data derived from national and state‐wide assessments, tailoring and augmenting existing health assessment instruments, ultimately leading to a smaller, more intense evaluation of the population of highest concern. Longitudinal evaluation of this smaller cohort then integrates assessments across the research continuum from basic (mechanistic) to clinical (biomarkers) to population‐based studies (implementation science) [42]. As we observed during the HEAL study and in the aftermath of many natural and technological disasters, the interconnectedness of chemical and non‐chemical stressors can impact health in a more pronounced fashion than either stressor alone. Hence, the importance of taking a multi‐system enterprise‐wide approach in comprehensively assessing the impact of disasters on vulnerable communities, especially those living in disaster‐prone regions [43].

## 4 Education

Educating future public health professionals about asthma, including its triggers and potential interventions, is essential for fostering community resilience. This section highlights two educational initiatives at Tulane University and the University of Pittsburgh that engage high school students in public health research and practice.

**Figure 2:**
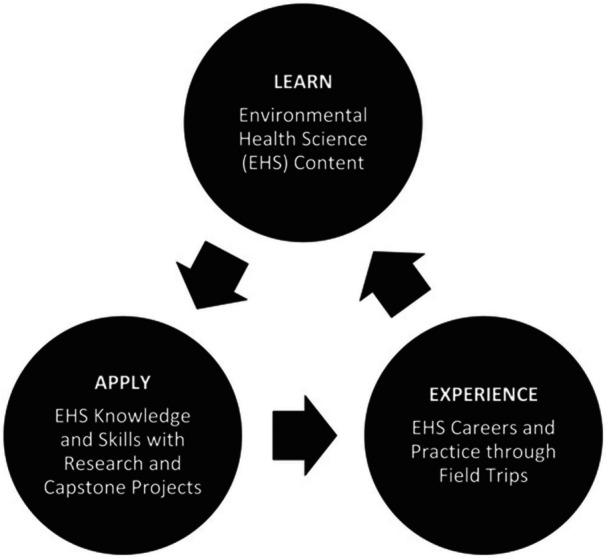
Environmental health sciences academy educational framework.

The Center for Gulf Coast Environmental Health Research, Leadership, and Strategic Initiatives at Tulane University launched the Emerging Scholars Environmental Health Sciences Academy (EHSA) in 2013. This summer program targeted public high school students from Louisiana, Florida, and Alabama, offering hands‐on experience in environmental health science. EHSA operated from 2013 to 2018, with 120 students participating in total. Its educational framework (Figure 2) emphasized experiential learning, which significantly increased students' motivation to pursue science and led many to enroll in college and pursue degrees in health sciences [44].

At the University of Pittsburgh School of Public Health, the Public Health Science Academy (PHSA) is a 4‐week summer program for current 10th and 11th‐grade students. Since its inception in 2022, PHSA has hosted 49 students from seven Pittsburgh‐area high schools [45]. Each summer, students select a research topic and collaborate with faculty mentors, culminating in a research symposium where they present posters and engage with the community. Projects span a wide range of public health issues, with several focusing on environmental health each year. Table 1 provides examples of asthma‐related research projects conducted between 2022 and 2025.

**Table 1:** Examples of asthma‐related research projects in the PHSA program.

Project title	Year
Early‐Life Exposure to Inorganic Arsenic Primes the Offspring to Increased Asthma Risk	2022
Air Quality: Particle Pollution and Health in Pittsburgh	2023
Extreme Heat and Air Pollution	2024
Air Pollution and Asthma in Allegheny County	2025

In July 2025, the University of Pittsburgh School of Public Health received the Justin Reid Ehrenwerth Community Impact Award from the Jewish Healthcare Foundation [46, 47]. This award supports a year‐long research initiative in which two PHSA students will intern with 3 Rivers Waterkeeper, a Pittsburgh‐based nonprofit dedicated to protecting the water quality of the Allegheny, Monongahela, and Ohio rivers and their watersheds [48]. Through this internship, students will gain firsthand experience in water quality monitoring and analysis within the local community.

Programs like EHSA and PHSA play a vital role in cultivating a knowledgeable and resilient public health workforce equipped to address the growing challenges of childhood asthma and environmental pollution.

## 5 Practice

### 5.1 Community Health Workers (CHWs) as Asthma Counselors

Preclinical identification of asthma triggers can be strengthened by integrating CHWs into care teams as dedicated asthma counselors. Following the tiered framework used in community health practice, CHWs can contribute at three levels of engagement [49]. Tier 1 CHWs (entry‐level) provide basic asthma trigger screening in clinics, schools, or community settings, asking families about common exposures such as pets, mold, dust, or smoking in the home. Tier 2 CHWs (intermediate) conduct structured home visits to observe housing conditions (ventilation, pests, dampness), assess outdoor exposures, and deliver tailored education to families about trigger reduction. Tier 3 CHWs (advanced), who have additional training in environmental health, collaborate directly with clinicians and public health agencies to coordinate remediation services, connect families to social support systems, and advocate for community‐level interventions such as improved housing policies or neighborhood air quality monitoring. Importantly, across all tiers, CHWs bring cultural and linguistic competence, build trust with families, and help families share their real, day‐to‐day realities that shape health, which may not come up in standard medical history or brief clinic visit. By embedding CHWs as asthma counselors, pediatric care teams can more effectively address environmental contributors to asthma, thereby reducing disparities and strengthening long‐term resilience in high‐risk populations.

## 6 Conclusion

Throughout the three case studies, we learned the following: childhood asthma exacerbations are resulting from multiple triggers, all directly linked to environmental conditions. Those conditions, in turn, result in exposures to complex mixtures of chemical and non‐chemical stressors. The origin of those stressors is multifactorial—from natural disasters to environmental contamination of manufacturing plants to technological accidents. Across the case studies, the role of psychosocial stress was not only common but played a key role in the overall health status of asthmatic children. Likewise, the extent of a community's resilience significantly contributes to mitigating environmentally induced asthma exacerbations regardless of the presence of genetic predisposition.

**Figure 3:**
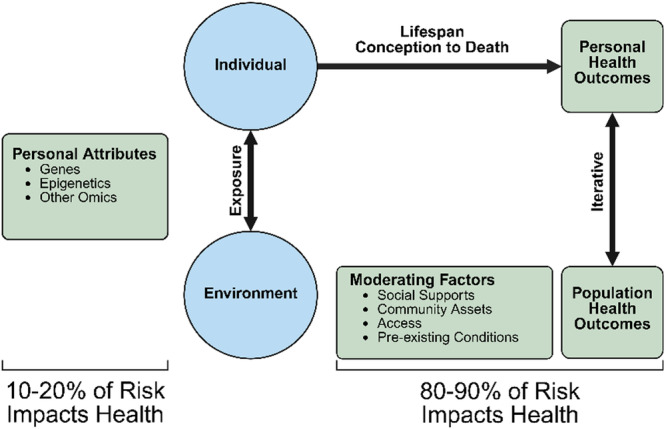
Influences on personal health outcomes.

Individual resilience is not enough to counter childhood asthma; the social determinants of health and the strength of the community capital play a key role in sustaining a positive health trajectory for children with asthma. As depicted in Figure 3, the magnitude of adverse health outcomes, especially exacerbation of childhood asthma, is predominantly influenced by community‐level moderators and environmental influences [50]. Research on ways communities and families of children with asthma can respond to environmental challenges will be a key to improving resilience.

Based on the lessons learned from these case studies, future childhood asthma environmental epidemiology research should assess exposures to mixtures of chemical and non‐chemical stressors, while taking into account the impact of community resilience determinants on potential interventions.

## Author Contributions

**Maureen Y. Lichtveld:** conceptualization, writing – original draft, writing – review and editing, supervision. **Kaitlin Kirkpatrick Heimke:** project administration, writing – review and editing, writing – original draft, visualization. **Laura J. Dietz:** writing – original draft. **Firoz Abdoel Wahid:** writing – original draft. **Jeanine M. Buchanich:** writing – original draft. **Brian Conner Earle:** writing – original draft, visualization. **Terry L. Noah:** conceptualization, writing – original draft.

## Funding

The authors received no specific funding for this work.

## Conflicts of Interest

The authors declare no conflicts of interest.

## Data Availability

The authors have nothing to report.
